# Inhibition of HIV-1 reactivation by a telomerase-derived peptide in a HSP90-dependent manner

**DOI:** 10.1038/srep28896

**Published:** 2016-07-01

**Authors:** Hong Kim, Myung-Soo Choi, Kyung-Soo Inn, Bum-Joon Kim

**Affiliations:** 1 Department of Microbiology and Immunology, Liver Research Institute, Biomedical Sciences and SNUMRC, College of Medicine, Seoul National University, Seoul, Korea; 2 Department of Pharmaceutical Science, College of Pharmacy, Kyung Hee University, Seoul, Korea

## Abstract

A peptide vaccine designed to induce T-cell immunity to telomerase, GV1001, has been shown to modulate cellular signaling pathways and confer a direct anti-cancer effect through the interaction with heat shock protein (HSP) 90 and 70. Here, we have found that GV1001 can modulate transactivation protein-mediated human immunodeficiency virus (HIV)-1 transactivation in an HSP90-dependent manner. GV1001 treatment resulted in significant suppression of HIV-1 replication and rescue of infected cells from death by HIV-1. Transactivation of HIV-long terminal repeat (LTR) was inhibited by GV1001, indicating that GV1001 suppressed the transcription from proviral HIV DNA. The anti-HIV-1 activity of GV1001 was completely abrogated by an HSP90-neutralizing antibody, indicating that the antiviral activity depends on HSP90. Further mechanistic studies revealed that GV1001 suppresses basal NF-κB activation, which is required for HIV-1 LTR transactivation in an HSP90-dependent manner. Inhibition of LTR transactivation by GV1001 suggests its potential to suppress HIV-1 reactivation from latency. Indeed, PMA-mediated reactivation of HIV-1 from latent infected cells was suppressed by GV1001. The results suggest the potential therapeutic use of GV1001, a peptide proven to be safe for human use, as an anti-HIV-1 agent to suppress the reactivation from latently infected cells.

Human reverse transcriptase subunit of telomerase (hTERT) is highly expressed in various cancer tissues and it has been considered as an attractive target for the development of effective cancer vaccines^1^. A peptide vaccine, encompassing the 16mer MHC class II epitope (611-EARPALLTSRLRFIPK-626) of hTERT, GV1001, has been developed as a therapeutic vaccine to induce T-cell immune responses^2^. Several phase I/II clinical trials have confirmed the safety and capability of inducing specific T-cell responses in patients with pancreatic cancer, non-small cell lung cancer (NSCLC), melanoma and hepatocellular carcinoma^3^,^4^,^5^,^6^,^7^. In addition, a clear positive correlation between induced immune responses and prolonged survival of advanced pancreatic cancer patients has been shown in a phase II study^3^.

In previous studies, we have shown that GV1001 interacts with extracellular heat shock protein 90 (HSP90) and penetrates into the cytoplasm of cells^8^. Moreover, GV1001 exerted a strong anti-cancer effect through the interaction with HSP90 under hypoxic conditions by modulating the HIF-1α-VEGF signaling axis^9^. These studies indicate that GV1001 can regulate intracellular signaling pathways through the interaction with HSP90.

HSPs are molecular chaperones, and they play crucial roles in maintaining protein homeostasis and cell homeostasis, particularly under stress conditions^10^. HSP90 has been associated with several pathological conditions such as cancer, atherosclerosis and virus infection^11^,^12^,^13^,^14^. The HSP90 client list includes numerous proteins related to tumorigenesis, invasiveness and metastasis^15^. Thus, HSP90 has emerged as a promising target for cancer therapeutics, and several HSP90 inhibitors have been developed and are undergoing clinical trials^16^. Interestingly, secreted HSP90 and cell surface HSP90 have been observed in cancer cells, and these extracellular HSP90 (eHSP90) proteins promote cancer growth and angiogenesis^17^,^18^. Noncancerous cells also produce eHSP90 under various environmental conditions, including heat, hypoxia and starvation^17^. eHSP90 plays distinct functions from those of intracellular HSP90, and it can regulate cell signaling pathways by interacting with various cell surface proteins^17^. Upon virus infection, robust production of viral proteins also requires HSP functions, and the list of viruses suppressed by HSP90 inhibitors continues to grow^19^. Recent studies have shown that human immune deficiency virus-1 (HIV-1) infection also resulted in increased expression of HSP90 in mononuclear cells and T-cells^20^,^21^. Indeed, HSP90 plays a pivotal role in HIV replication by acting at multiple steps of the life cycle of the virus. HSP90 involvement in HIV viral transcription and HIV replication in acutely infected cells was suppressed by HSP90 inhibitors^22^. Moreover, HSP90 regulates HIV reactivation from latency by modulating NF-κB signaling^23^.

Considering that GV1001 interacts with HSP90 and modulates cell signaling, we explored the possible antiviral role of GV1001 against HIV-1 in the current study.

## Results

### GV1001 suppresses HIV-1 replication

**Figure 1:**
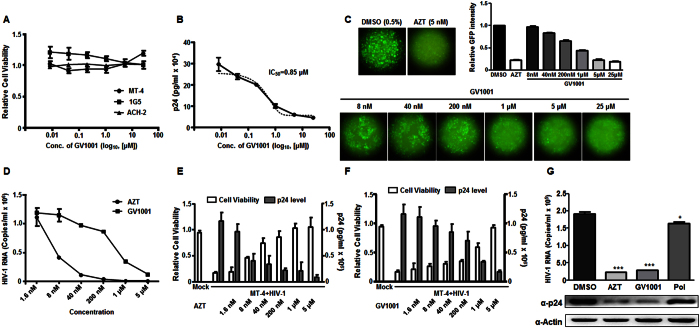
Inhibition of HIV-1 replication by GV1001. (**A**) Effect of GV1001 on cell viability. MT-4, 1G5 and ACH-2 cells were treated with increasing concentrations of GV1001 for 5 days and subjected to the MTT assay. (**B**) Effect of GV1001 on HIV-1 virus production. MT-4 cells were infected with HIV-1 and treated with increasing concentrations of GV1001. Amounts of viral particles in the supernatants were determined by p24 ELISA. (**C**) Effect of GV1001 on the expression of eGFP, which is under the same control as HIV-1 Nef expression. MT-4 cells infected with HIV-1 were treated with increasing concentrations of GV1001, and the expression of eGFP was monitored by fluorescence microscopy and quantitated. (**D**) Inhibition of HIV-1 virus particle production. MT-4 cells were infected with HIV-1 and treated with increasing concentrations of AZT or GV1001. The levels of viral genomes in the supernatants were determined by RT-qPCR as described in the Materials and Method. (**E,F**) Protection of cells from HIV-1 infection-mediated cell death by GV1001. MT-4 cells (1 × 10^4^ cells) were infected with HIV-1 virus (4 × 10^5^ CCID_50_) and treated with AZT (**E**) or GV1001 (**F**) for 5 days and subjected to the cell viability assay and p24 ELISA. Data represent means ± SD. (**G**) Inhibition of HIV-1 viral RNA synthesis and p24 production by GV1001 in HIV-1 infected PBMCs. PBMCs were infected with HIV-1 and treated with DMSO (0.5%), AZT (5 nM), GV1001 (10 μM), or HBV Pol-derived control peptide (Pol, 10 μM). Levels of viral mRNA and p24 protein were determined by RT-qPCR and immunoblotting assay. Data represent means ± SD. *p < 0.05 versus DMSO, ***p < 0.001 versus DMSO.

Prior to examination of the role of GV1001, we analyzed the cell cytotoxicity of GV1001 to exclude the possibility that GV1001 affects the replication of HIV-1 due to its nonspecific cell cytotoxicity. GV1001 does not exert significant cytotoxic activity against MT-4, IG5 and ACH-2 cells up to 25 μM (Fig. 1A). First, the anti-HIV-1 activity of GV1001 was determined by analyzing its effect on HIV-1 (pBR_HIV-1-M-NL4-3_IRES_eGFP) replication in MT-4 cells. As determined by p24 ELISA, production of viral particles in MT-4 cells was significantly inhibited by GV1001 in a dose-dependent manner, and the mean 50% inhibitory concentration (IC_50_) value was approximately 0.85 μM (Fig. 1B). Additionally, eGFP production, which depends on the activation of HIV-1 LTR, was also diminished by treatment with GV1001 (Fig. 1C). Inhibition of viral particle production by GV1001 was further confirmed by determining the HIV-1 genomic RNA levels of produced viral particles. GV1001 showed a dose-dependent suppressive effect (Fig. 1D). A peptide derived from HBV polymerase did not exert any significant effect on HIV virion production and eGFP production, indicating that the anti-HIV function of GV1001 is not non-specific (Fig. S1).

In line with the suppression of HIV-1 replication by GV1001, the latter displayed a cell protective effect on HIV-1-infected MT-4 cells. As depicted in Fig. 1E,F, AZT and GV1001 showed a significant cell protective effect in a dose-dependent manner. Similar to AZT, 5 μM GV1001 was sufficient to confer almost 100% cell protection from HIV-1-mediated cell death. Its cell protective effect was inversely correlated with a decreased supernatant p24 level, suggesting that GV1001 can protect cells by suppressing viral replication. Furthermore, GV1001 treatment resulted in decreased intracellular HIV viral genomic RNA, p24 protein synthesis and eGFP expression in human peripheral blood mononuclear cells (PBMCs) infected with HIV-1 (Figs 1G and S2).

### HIV-1 transcription is inhibited by GV1001

**Figure 2:**
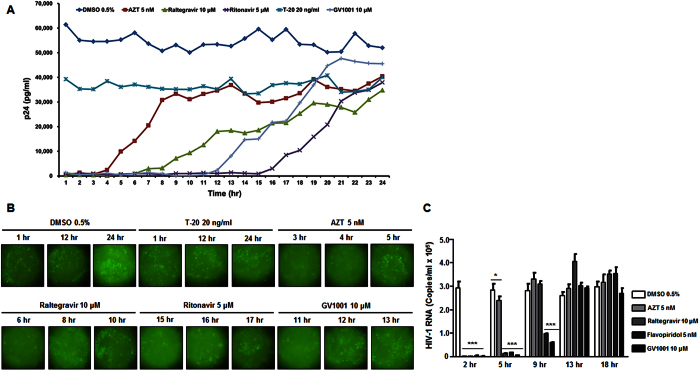
Inhibition of HIV-1 replication by GV1001 at the transcriptional level. (**A**) Time-of-addition assay. Designated anti-HIV-1 agents, including GV1001, were used to treat HIV-1-infected MT-4 cells at different time points after infection. HIV-1 replication was assessed by p24 ELISA 5 days after HIV-1 infection. (**B**) Representative eGFP images from (**A**). (**C**) Inhibition of HIV-1 viral mRNA synthesis by GV1001. MT-4 cells were infected with HIV-1, and vehicle or antiviral agents were treated at the indicated time points. Levels of viral mRNA were determined by RT-qPCR. Data represent means ± SD. *p < 0.05 versus DMSO, ***p < 0.001 versus DMSO.

Considering that eGFP production from the HIV-1 genome is under the same control as Nef, decreased expression of eGFP in HIV-1-infected cells by GV1001 indicates the suppression of HIV-1 transcription by GV1001 (Fig. 1C). To further delineate the underlying mechanism of HIV-1 inhibition by GV1001, a time-of-addition (TOA) study was performed using GV1001 and several anti-HIV drugs covering different stages of HIV replication. The results of each reagent in the TOA assay have shown that the inhibition of HIV replication was well represented to a time point corresponding to the occurrence of the replication step targeted by the drugs, and GV1001 started to lose its activity at the time point between 11 hours and 13 hours after HIV-1 infection (Fig. 2A). Analysis of eGFP expression confirmed that the inhibitory activity of GV1001 is weakened when treated for 12 hours after infection (Fig. 2B). According to the typical result from a time-of-addition assay, the viral transcription of HIV from the integrated HIV genome occurs from 11 to 13 hours after infection^24^. Thus, this result suggests that the mode of action of GV1001 in the MT-4 cells infected with HIV is to inhibit HIV proliferation through suppressing the transcription activity. Furthermore, GV1001 efficiently suppressed the production of the viral mRNA of HIV-1 when cells were treated with it for up to 9 hours after infection, while it lost its activity to lower the viral mRNA when it was treated 13 hours after infection (Fig. 2C). At the same time, there was no significant change in the housekeeping host GAPDH mRNA synthesis, suggesting that GV1001 specifically regulates HIV-1 viral transcription.

### GV1001 inhibits Tat-dependent HIV-1 transcription

**Figure 3:**
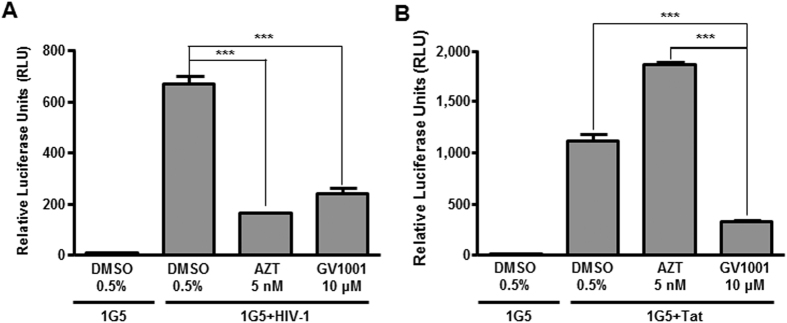
Suppression of Tat-dependent HIV-1 transcription by GV1001. (**A**) 1G5 cells, which harbor a stably integrated HIV-LTR-luciferase construct, were infected with HIV-1, followed by treatment with DMSO, AZT or GV1001. Four days after infection, cell lysates were subjected to luciferase assay to analyze the transactivation of HIV-LTR. (**B**) 1G5 cells were transfected with the Tat plasmid. Twelve hours after transfection, cells were treated with vehicle (DMSO), AZT or GV1001 as indicated. Four days after transfection, HIV-LTR transactivation was analyzed by the luciferase assay. Data represent means ± SD. ***p < 0.001.

HIV-1 transactivating protein (Tat) enhances HIV-1 transcription through the interaction with the tat-transactivation-responsive region (TAR). Because GV1001 is likely to specifically regulate HIV-1 transcription, we have tested whether GV1001 affects the transactivating role of HIV-1 Tat. We exploited 1G5, which is a Jurkat derivative cell line containing a stably integrated HIV-LTR-luciferase construct. After 1G5 cells were infected with HIV-1 or transduced with a tat-retroviral vector (pSV2tat72) in the presence of AZT and GV1001, luciferase activity was analyzed. 1G5 cells infected with HIV-1 showed a dramatic increase in luciferase activity, and AZT or GV1001 treatment significantly reduced the effect of HIV-1 infection on HIV-LTR-Luciferase activity by fivefold (Fig. 3A). Consistently, GV1001 suppressed the activation of HIV-LTR luciferase activity induced by ectopic expression of Tat, suggesting its capability to regulate the transactivating role of tat during HIV-1 infection (Fig. 3B).

### GV1001 suppresses the reactivation of HIV-1 from latency

**Figure 4:**
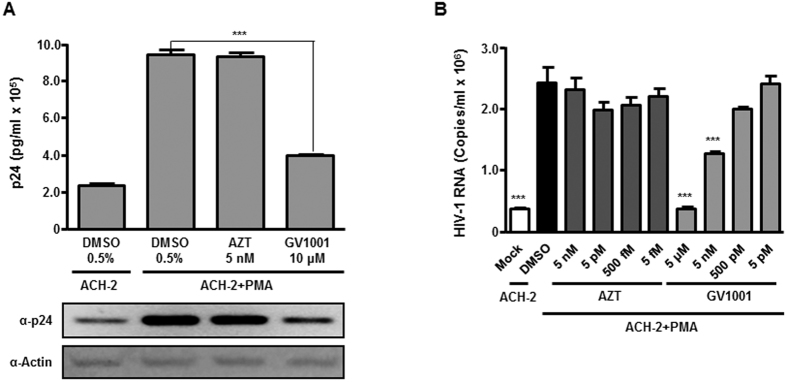
Suppressive effect of GV1001 on reactivation of HIV-1 from latency. (**A**) ACH-2 cells, which are latently infected with HIV-1, were stimulated with PMA (50 nM) to induce the reactivation of HIV-1 for 1 hour. Next, cells were treated with DMSO, AZT or GV1001 for 24 hours. The level of produced viral particles in the supernatants was determined by p24 ELISA. (**B**) ACH-2 cells were treated with PMA followed by treatment with increasing concentrations of AZT or GV1001 as in (**A**). The levels of produced viral particles were determined by measuring the amount of viral genomic RNAs using RT-qPCR. Data represent means ± SD. ***p < 0.001 versus DMSO control.

Given that GV1001 regulates Tat-dependent transcriptional activity, the role of GV1001 in HIV-1 reactivation was investigated. ACH-2 cells, a human T cell line encompassing a single copy of proviral HIV-1 DNA, were treated with PMA along with vehicle, AZT or GV1001. PMA treatment significantly increased the supernatant p24 level, and GV1001 almost abolished the effect, whereas AZT did not change the effect of PMA (Fig. 4A). This result indicates that GV1001 suppresses the PMA-induced HIV-1 reactivation and production of viral particles. Similarly, HIV-1 viral RNA genome levels were also significantly decreased in the supernatants from PMA-treated cells following treatment with GV1001 in a dose-dependent manner, supporting the previous data (Fig. 4B).

### Anti-HIV-1 activity of GV1001 is HSP90-dependent

**Figure 5:**
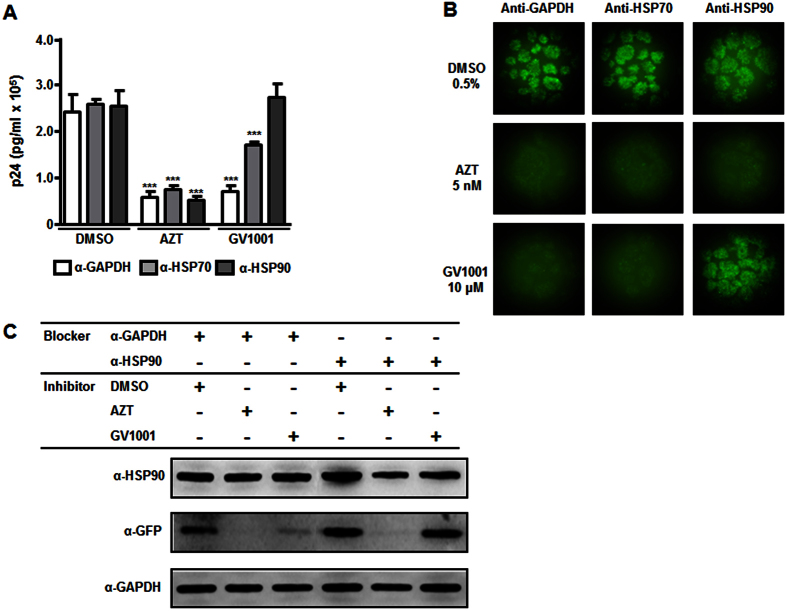
Crucial role of HSP90 in the anti-HIV-1 activity of GV1001. (**A**) MT-4 cells were infected with HIV-1 for 1 hour and then treated with anti-GAPDH, anti-HSP70 or anti-HSP90 antibodies 1 hour prior to treatment with DMSO, AZT or GV1001. Twenty-four hours after infection, the production of HIV-1 particles was determined by p24 ELISA. Data represent means ± SD. ***p < 0.001 versus DMSO control. (**B**) Representative eGFP images from (**A**). (**C**) MT-4 cells were infected with HIV-1 and treated with anti-GAPDH or anti-HSP90 antibody. Cells were treated with DMSO, AZT or GV1001 for 24 hours as indicated. Cell lysates were analyzed by immunoblotting to examine the expression of eGFP.

Next, it has been examined whether GV1001 modulates HIV-1 replication via its interaction with HSPs. Surprisingly, GV1001-mediated suppression of HIV-1 production in MT-4 cells was completely restored by treatment with an anti-HSP90 neutralizing antibody, while AZT-mediated suppression was not affected at all (Fig. 5A). Anti-HSP70-neutralizing antibody treatment resulted in partial restoration, and an isotype control anti-GAPDH antibody showed no significant effect (Fig. 5A). Suppression of eGFP expression, which depends on HIV-1 transcriptional activity by GV1001, was also reverted by the anti-HSP90 antibody, whereas the effect of AZT was not affected (Fig. 5B,C). These results show that GV1001 can regulate HIV-1 transcriptional activity through its interaction with HSP90.

### GV1001 suppresses basal NF-κB transcriptional activity

**Figure 6:**
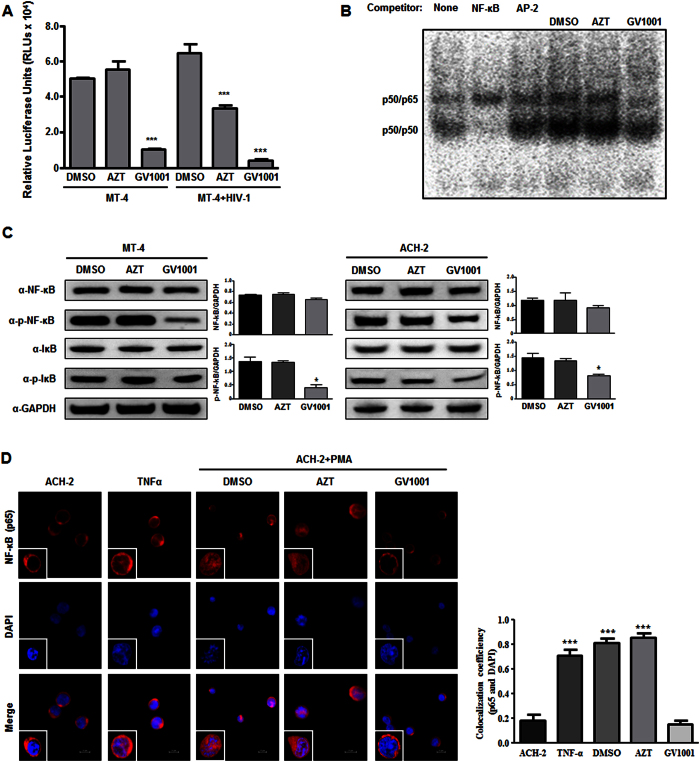
Inhibition of basal NF-kB activation by GV1001. (**A**) MT-4 cells were transfected with NF-κB firefly luciferase and CMV-promoter Renilla luciferase reporter plasmids. Next, cells were infected with HIV-1 (1 × 10^6^ CCID_50_) and treated with the designated compounds for 24 hours, followed by dual-luciferase assay. Data represent means ± SD. ***p < 0.001 versus DMSO control. (**B**) MT-4 cells were infected with HIV-1 and treated with DMSO, AZT or GV1001 as indicated. Twenty-four hours after infection, the nuclear fractions were extracted and subjected to EMSA assay. NF-κB and AP-2 competitor oligomers were used to confirm the specificity. (**C**) MT-4 cells and ACH-2 cells were treated with DMSO, AZT (5 nM) or GV1001 (10 μM) for 1 hour. Cell lysates were subjected to immunoblotting analysis using indicated antibodies. (**D**) ACH-2 cells were stimulated with TNF-α (30 ng/ml) or PMA (50 nM) for 1 hour and treated with DNSO, AZT or GV1001 for 24 hours. Next, cells were stained with anti-p65 NF-κB antibody and Alexa Flour 594-conjugated secondary antibody after permeabilization. After brief DAPI nuclear staining, cells were observed by confocal microscopy. Colocalization coefficiency of p65 and DAPI was determined by Manders’ overlap coefficient analysis using NIS-Elements Ver 4.2 image software. Scale bar, 10 μm. Data represent means ± SD. ***p < 0.001 versus ACH-2 control.

**Figure 7:**
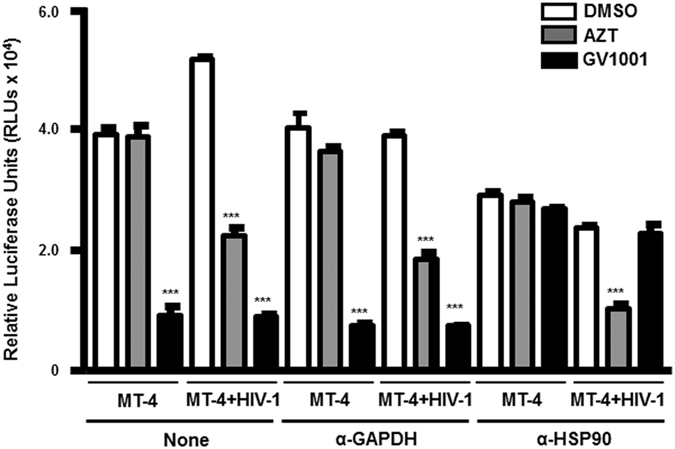
Crucial role of HSP90 in the GV1001 suppressive effect on basal NF-κB activity. MT-4 cells were transfected with NF-κB firefly luciferase and CMV-promoter Renilla luciferase reporter plasmids. Next, cells were treated with designated antibodies (10 ng/ml) 1 hour prior to HIV-1 infection. After HIV-1 infection, cells were treated with DMSO, AZT or GV1001 for 24 hours and subjected to the dual-luciferase assay. Data represent means ± SD. ***p < 0.001 versus DMSO control.

Given that NF-κB can trigger HIV transcription by interacting with HIV-LTR, we tested the hypothesis that GV1001 modulates NF-κB activity in an HSP90-related manner to regulate HIV-1 transcriptional activity. Treatment with GV1001 resulted in a dramatic decrease in basal NF-κB activity regardless of HIV-1 infection in MT-4 cells (Fig. 6A). By contrast, AZT showed no significant effect on NF-κB activity in MT-4 cells. AZT showed a moderate suppressive effect in MT-4 cells infected with HIV-1, a finding that might be due to the low level of HIV replication (Fig. 6A). The suppressive effect on basal NF-κB activity of GV1001 was further confirmed by EMSA. As shown in Fig. 6B, GV1001-treated cells showed clear reduction of p65 NF-κB activation, indicating that GV1001 suppresses the basal level of NF-κB DNA binding in the nucleus. GV1001 treatment also leads to reduced NF-κB (p65) phosphorylation, indicating that GV1001 suppresses the cytosolic activation of NF-κB and subsequent nuclear translocation (Fig. 6C). A similar result was obtained from ACH-2 cells latently infected with HIV-1 (Fig. 6C). As expected, treatment with GV1001 resulted in decreased nuclear localization of NF-κB (p65) in PMA-treated ACH-2 cells compared with DMSO-treated control cells (Fig. 6D). Because we have shown that the anti-HIV effect of V1001 depends on HSP90, we tested whether the NF-κB suppressive effect is dependent on HSP90. Consistent with the anti-HIV activity data, the NF-κB suppressive activity of GV1001 was also completely reversed by treatment with an HSP90 blocking antibody or HSP90 inhibitor, while treatment with an anti-GAPDH antibody showed no significant effect (Fig. 7).

## Discussion

Previously, we have shown that GV1001 interacts with extracellular HSP90 and is internalized into cells. In Hepatitis C virus (HCV)-infected cells, GV1001 suppressed the replication of HCV through the interaction with HSP90^8^,^25^. Given that HSP90 plays crucial roles in the HIV-1 life cycle and that GV1001 interacts with HSP90, it was very tempting to examine the possible antiviral activity of GV1001 against HIV-1. To our surprise, GV1001 displayed potent anti-HIV-1 activity. Our Time-of-addition study suggested that GV1001 suppresses the replication of HIV-1 at the level of viral transcription. Indeed, GV1001 suppressed TAT-dependent transcriptional activity and decreased the level of HIV-1 RNA (Figs 2C and 3A,B). Inhibition of TAT-dependent viral transcription by GV1001 suggests that it might be able to suppress the reactivation of latently infected HIV-1. Despite the successful suppression of HIV replication by highly active antiretroviral therapy (HAART), current therapeutic strategies cannot eradicate latently infected HIV-1, and reactivation of the virus is a cause of treatment failure^26^,^27^. Because GV1001 has already been proven to be safe by several clinical trials, it is worthwhile to further evaluate the potential use of GV1001 as an anti-HIV agent to suppress the reactivation. As expected, GV1001 efficiently suppressed the HIV-1 reactivation from latently infected cells, suggesting its potential use as a therapeutic agent.

Of note, treatment with an anti-HSP antibody resulted in the loss of anti-HIV-1 activity of GV1001 suggesting that the anti-HIV-1 activity of GV1001 is largely due to its interaction with HSP90. Since antibodies against HSP90 cannot penetrate cell membranes and interact with intracellular HSP90, the result suggests that GV1001 interact with cell surface HSP90 and modulates HIV-1 transcription. Although the partial inhibition of anti-HIV-1 function of GV1001 by an anti-HSP70 antibody suggests that HSP70 also be participated in the suppression by GV1001, complete abrogation of the GV1001 activity by the anti-HSP90 antibody treatment indicates that HSP90 is the major factor. Recently, it has been shown that cell surface HSP90 serves as a co-factor for NF-κB activation and involved in cellular pathogenesis by latently infected Kaposi’s sarcoma-associated herpesvirus (KHSV)^28^. In addition, several studies have shown that eHSP90 regulates several cellular signaling pathways, including the NF-κB pathway^29^. NF-κB can trigger HIV transcription by interacting with NF-κB binding sites within HIV-LTR, and it enhances TAT-mediated LTR transactivation^30^. In addition, Tat can directly activate NF-κB^31^. We showed that treatment with GV1001 resulted in suppression NF-κB activation and subsequent down-regulation of TAT-dependent transcription. Moreover, suppression of NF-κB activation by GV1001 was restored by the treatment with an anti-HSP90 antibody.

Collectively, we show that GV1001 suppresses the basal level of NF-κB activity, leading to the suppression of HIV-LTR transactivation. Although the underlying molecular mechanisms of NF-κB suppression remain elusive, the results clearly indicate that HSP90 is implicated in the activity. An elegant previous study showed that intracellular HSP90 plays a crucial role in HIV reactivation by directly regulating NF-κB^23^. In the current study, nullification of the GV1001 effects by an anti-HSP90 antibody strongly suggests that eHSP90 or cell surface HSP90 may also be involved in NF-κB signaling and HIV-LTR activation. Indeed, recent studies have shown that extracellular HSP90 can enhance the NF-κB signaling pathway in various cell types^28^,^32^,^33^. Investigating the roles of extracellular HSP90 in NF-κB signaling and HIV replication in T-cells are currently underway.

## Materials and Methods

### Peptides and Reagents

A 16-amino acid human telomerase reverse transcriptase (hTERT)-derived peptide, GV1001, and a 13-amino acid HBV polymerase derived control peptide (Pol-LQHGRLVFQTSTR) were synthesized and characterized as described previously^34^. T-20, Raltegravir, Flavopiridol, and Rinonavir were obtained from the NIH/AIDS Research and Reference Reagent Program (NIH, Bethesda, MD). Azidothymidine (3-Azido-3-deoxythymidine, AZT) was purchased from Sigma-Aldrich (St. Louis, MO). Antibodies against HSP90 (#4877S), phospho-NF-κB (p65, #3033S), IκB (#4814S) and phopho-IκB (#2859S) were obtained from Cell Signaling (Cell Signaling, Danvers, MA), and anti-p24 antibody (Ab9071) was bought from Abcam (Abcam, Cambridge, MA). Antibodies against HSP70 (sc32239), GFP (sc81045), GAPDH (sc25778) and NF-kB (p65, sc372) were purchased from Santa Cruz Biotechnology (Santa Cruz, Dallas, TX).

### Cells, plasmids and virus

The human T-cell leukemia cell line MT-4, the ACH-2 cell line latently infected with HIV-1, and a Jurkat derivative 1G5 cell line containing a stably integrated HIV-LTR-luciferase construct were obtained from the NIH/AIDS Research and Reference Reagent Program. The pSV2tat72 plasmid (Cat. No. 294, provided by Dr. Alan Frankel) producing Tat protein (residues 1–72) was obtained from the NIH/AIDS Research and Reference Reagent Program^35^. To produce HIV-1, 293FT cells were transfected with the pBR_HIV-1_M_NL4-3_IRES_eGFP vector which co-expresses Nef and enhanced green fluorescence protein (eGFP) from a single bicistronic RNA (Cat. No. DS441, provided by Dr. Daniel Sauter and Dr. Frank Kirchhoff) using Lipofectamine 2000 reagent (Life Technologies)^36^. Forty-eight hours after transfection, virus-containing medium was harvested and the viral titer was determined using p24 ELISA (ABL, city, MD). For amplification of infectious HIV-1, MT-4 cells were infected with generated HIV-1 (MOI = 0.5) for 48 hours. After brief centrifugation, the supernatant was filtered and subjected to titration using p24 ELISA.

### Anti-viral effect assay

To evaluate the anti-HIV-1 effect of GV1001, the cell-based anti-viral effect assay was performed. MT-4 cells (4 × 10^5^ cells) were infected with HIV-1 (4 × 10^5^ CCID_50_) for 1 hour. After washing, infected cells were seeded and treated with GV1001 or anti-HIV-1 drugs. After 2 days of incubation, the images of MT-4 cells expressing EGFP were obtained using fluorescence microscopy. The relative GFP intensity was determined using ImageJ software program (National Institutes of Health) in pixels per area. The collected supernatant was subjected to p24 ELISA or RNA extraction for reverse transcription-quantitative polymerase chain reaction (RT-qPCR) to determine the extracellular viral amount. The cell pellets were used for the cell viability assay. To examine the role of HSP90 in the antiviral action of GV1001, MT-4 cells were infected with HIV for 1 hour and treated with anti-HSP70 (10 ng) or anti-HSP90 (10 ng). HIV-LTR-dependent synthesis of eGFP was also confirmed by immunoblotting using an anti-GFP antibody with cell lysates. For antiviral assays using normal human PBMCs, separated PBMCs were activated with 1 μg/ml phytohemaggulutinin (PHA) (Sigma-Aldrich, St. Louis, MO) for 3 days in the presence of IL-2 (100 U/ml) (PEPROTECH, Rocky Hill, NJ). PHA-stimulated PBMCs were infected with HIV-1 at a multiplicity of infection (MOI) of 0.1 and treated with indicated reagents for 3 days and subjected to fluorescent microscopy. To determine the viral replication in PBMCs, intracellular viral RNAs were isolated and analyzed by RT-qPCR as described below. All studies were performed following protocols approved by the Seoul National University Hospital Institutional Review Board (IRB, No 1605-056-761).

### Cell cytotoxicity assay

MT-4, 1G5 or ACH-2 cells were seeded (1 × 10^4^ cells/well) in 96-well microplates and incubated with increasing concentrations of GV1001 for 5 days. Cell viability was determined using CellTiter96 kit (Promega, WI). To analyze the cell protective effect of GV1001 from HIV-1-induced cell death, MT-4 cells (1 × 10^4^ cells) were infected with HIV-1 virus (4 × 10^5^ CCID_50_) for five days with or without GV1001 and subjected to cell viability assays.

### Determination of HIV-1 virus production

To measure HIV-1 viral titers, HIV-1 p24 antigen capture ELISA (p24 ELISA, ABL) and RT-qPCR assay were conducted. ELISA was performed according to the manufacturer’s protocol. HIV-1 RNA genomes from cell culture supernatants and pellets were purified using the QIAamp Ultrasens Virus kit (Qiagen, Hilden, Germany) and quantitated by RT-qPCR using a primer pair specific to gag of HIV-1^31^. Glyceraldehyde phosphate dehydrogenase (GAPDH) was used as a reference gene for normalization^37^. The following primer pairs were used for real time qPCR: GagF, GCAGCCATGCAAATGTTAAAAGAG (sense) and GagR, TCCCCTTGGTTCTCTCATCTGG (antisense); and GAPDH-F, AATCCCATCACCATCTTCCA (sense) and GAPDH-R, TGGACTCCACGACGTACTCA (antisense). The HIV type 1 Genesig Standard kit (Primer design, Southampton, UK) was used to calibrate the viral titers.

### HIV-LTR transactivation assay

1G5 cells harboring an HIV-LTR luciferase reporter system were used to analyze the TAT-mediated transactivation of HIV-LTR. Cells were transfected with pSV2tat72 plasmid by electroporation or infected with HIV (1 × 10^5^ CCID_50_) along with polybrene (final concentration: 4 μg/ml). Then, cells were treated with vehicle (DMSO), AZT or GV1001 as indicated followed by further incubation for 4 days. Cell lysates were subjected to the luciferase assay, and LTR-dependent luciferase production was analyzed using a luciferase assay kit (Promega, WI).

### Analysis of the HIV-1 reactivation and virus production

To induce HIV reactivation from ACH-2 cells, 50 nM phorbol myristyl acetate (PMA) was added to cells for 1 hour. After washing with D-PBS twice, ACH-2 cells were seeded at a concentration of 1 × 10^5^ cells/ml in 48-well plates. Increasing concentrations of AZT or GV1001 were added to cells and further incubated for 24 hours. The collected supernatants were subjected to p24 ELISA to determine the extracellular viral amounts.

### Analysis of NF-κB activation

To investigate the effect of GV1001 on NF-κB signaling in MT-4 cells, the NF-κB luciferase assay and electromobility shift assay (EMSA) were utilized. For the NF-kB luciferase assay, MT-4 cells were transfected with NF-kB firefly luciferase plasmid together with the CMV-promoter Renilla luciferase reporter plasmid by electroporation and infected with HIV (1 × 10^6^ CCID_50_), followed by treatment with the designated compounds. Twenty-four hours after infection, cells were harvested and subjected to the Dual-luciferase assay. For EMSA analysis, MT-4 cells (1 × 10^6^ cells) were infected with HIV (1 × 10^5^ CCID_50_) for 1 hour and incubated with DMSO, AZT and GV1001 as indicated. Cells were harvested at 24 hours post-infection, and the nuclear fractions were extracted, followed by EMSA using the Gel Shift Assay system (Promega, Madison, WI). Briefly, NF-κB-specific oligonucleotide (5′-AGT TGA GGG GAC TTT CCC AGG C-3′) probes were radiolabeled with [γ-^32^P]ATP (3,000 Ci/mmol at 10 mCi/ml) and separated from unlabeled [γ-^32^P]ATP. In super-shift and competition assays, nuclear extract was incubated with unlabeled competitor (NF-κB oligo) and noncompetitor (AP-2 oligo, 5′-GAT CGA ACT GAC CGC CCG CGG CCC GT-3′) oligonucleotides (1.75 pmol) before adding [γ-^32^P]ATP-labeled NF-κB probe for 10 minutes at room temperature. Nuclear protein (10 μg) was incubated with [γ-^32^P]ATP-labeled NF-κB probe (20,000 cpm) for 20 minutes at room temperature and the reaction mixtures were analyzed using 6% DNA retardation gel separation and autoradiography. Activation of NF-κB was also analyzed by immunoblotting and confocal microscopy. For immunoblotting analysis, MT-4 cells and ACH-2 cells were treated with vehicle (DMSO, 0.5%), AZT (1 nM) or GV1001 (10 μM) for 24 hours, and the lysates were subjected to immunoblotting using anti-NF-κB and anti-pNF-κB (p65) antibodies. Relative band intensities were analyzed by ImageJ software. For confocal analysis, ACH-2 cells were incubated with DMSO (0.5%), AZT (1 nM) and GV1001 (10 μM) for 24 hours after stimulation with PMA (50 nM) and hTNF-alpha (30 ng/ml) for 1 hour. After fixation with 4% paraformaldehyde and permeabilization with 0.1% Triton-X-100, the cells were stained with anti-NF-kB (p65) antibody and secondary Alexa Flour 594-conjugated antibody. After brief nuclear staining with DAPI, cells were observed under a laser scanning confocal microscope (Nikon A1, Japan). Scale bar, 10 μm. Colocalization of p65 and DAPI was determined by Manders’ overlap coefficient analysis using NIS-Elements Ver 4.2 image software^38^.

### Statistical analysis

Statistical comparisons between the control and treated groups were analyzed using the Student’s *t*-test. The level of statistical significance was set at either *p* < 0.05 (*), 0.01 (**), or 0.001 (***). All experiments were independently repeated three times.

## Additional Information

**How to cite this article**: Kim, H. *et al*. Inhibition of HIV-1 reactivation by a telomerase-derived peptide in a HSP90-dependent manner. *Sci. Rep.* **6**, 28896; doi: 10.1038/srep28896 (2016).

## Supplementary Material

Supplementary Information
