# Supplementary material for: Inhibition of HIV-1 reactivation by a telomerase-derived peptide in a HSP90-dependent manner
Source: Sci Rep. 2016 Jul 1;6:28896. doi: 10.1038/srep28896 (PMC4929463; doi:10.1038/srep28896)
Supplement: Supplementary Information [file srep28896-s1.doc]

Supplementary Information

**Inhibition of HIV-1 reactivation by a telomerase-derived peptide in a HSP90-dependent manner**

**Hong Kim, Myung-Soo Choi, Kyung-Soo Inn, Bum-Joon Kim**

**Materials and methods**

**Antiviral effects of GV1001 against HIV-1.** MT-4 cells (4 × 10^5^ cells) were infected with HIV-1 (4 × 10^5^ CCID_50_) for 1 hour. After washing, infected cells were seeded and treated with AZT, GV1001 or a 13-mer amino acid peptide derived from Hepatitis B virus **(**HBV) polymerase (Pol-LQHGRLVFQTSTR). After 2 days of incubation, the images of MT-4 cells expressing EGFP were obtained using fluorescence microscopy. The relative GFP intensity was determined using ImageJ software program (National Institutes of Health) in pixels per area. The collected supernatant was subjected to RNA extraction for reverse transcription-quantitative polymerase chain reaction (RT-qPCR) to determine the extracellular viral amount.

**Inhibition of HIV-1 transcription by GV1001 in PBMC.** Human peripheral blood mononuclear cells (PBMC) were separated using Biocoll (BIOCHROME, Berlin Germany), grown in RPMI1640 medium supplemented with 10% FBS, and activated with 1 μg/ml phytohemaggulutinin (PHA) (Sigma-Aldrich, St. Louis, MO) for 3 days in the presence of IL-2 (100U/ml) (PEPROTECH, Rocky Hill, NJ). PHA-stimulated PBMC cells were infected with HIV-1 at a multiplicity of infection (MOI) of 0.1 and treated with reagents for 3 days. The expression of eGFP was monitored by fluorescence microscopy. All studies were performed following protocols approved by the Seoul National University Hospital Institutional Review Board (IRB, No 1605-056-761).

**
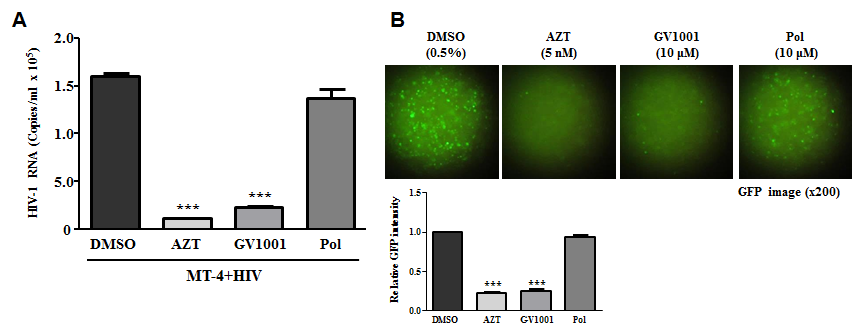
**

**Fig. S1 Anti-HIV-1 activity of GV1001.** (**A**) Effect of GV1001 on HIV-1 viral replication. MT-4 cells were infected with HIV-1 for 1 hour and treated with DMSO (0.5%), AZT (5 nM), GV1001 (10 μM), or a Pol (10 μM). Two days after treatment, the amount of HIV-1 genomic RNAs was determined by RT-qPCR. Data represent means ± SD. *** *p* < 0.001 versus DMSO. (**B**) Effect of GV1001 on the expression of eGFP. MT-4 cells infected with HIV-1 were treated with DMSO (0.5%), AZT (5 nM), GV1001 (10 μM), or a Pol (10 μM), and the expression of eGFP was monitored by fluorescence microscopy. The relative eGFP signal intensity was quantified using ImageJ software. Data represent means ± SD. *** *p* < 0.001 versus DMSO.

**
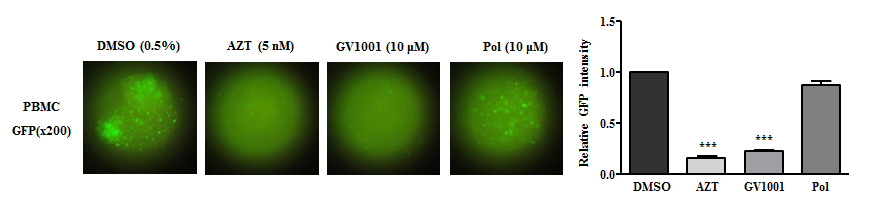
**

**Fig. S2 Inhibition of HIV-1 transcription by GV1001 in PBMC.** Effect of GV1001 on HIV-1 virus production in PBMC. PBMC cells were infected with HIV-1 at a multiplicity of infection (MOI) of 0.1 and trated with DMSO (0.5%), AZT (5 nM), GV1001 (10 μM), or a Pol (10 μM) for 3 days. After infection and treatment, the expression of eGFP was monitored by fluorescence microscopy. Relative eGFP signal intensity was quantified using ImageJ software. Data represent means ± SD. *** *p* < 0.001 versus DMSO.
